# Examination of stabilization of sedation by Nasal High Flow in patients with endoscopic retrograde cholangiopancreatography during sedation using Dexmedetomidine

**DOI:** 10.1097/MD.0000000000034004

**Published:** 2023-06-09

**Authors:** Takao Ayuse, Shinji Kurata, Tomotaka Mori, Shohei Kuroda, Taiga Ichinomiya, Rintaro Yano, Gaku Mishima, Eisuke Ozawa, Stanislav Tatkov, Shuntaro Sato, Nakao Kazuhiko, Tetsuya Hara

**Affiliations:** a Division of Clinical Physiology, Department of Translational Medical Sciences, Nagasaki University Graduate School of Biomedical Sciences, Nagasaki, Japan; b Department of Gastroenterology and Hepatology, Nagasaki University Graduate School of Biomedical Sciences, Nagasaki, Japan; c Nagasaki University Hospital, Clinical Research Center, Nagasaki, Japan; d Department of Anesthesiology and Intensive Care Medicine, Nagasaki University Graduate School of Biomedical Sciences, Nagasaki, Japan; e Department of Dental Anesthesiology, Nagasaki University Hospital, Nagasaki, Japan; f Fisher & Paykel Healthcare Ltd, Auckland, New Zealand.

**Keywords:** hypercapnia, intravenous anesthesia, Nasal High Flow

## Abstract

Dexmedetomidine is used for the sedation method in the case of endoscopic retrograde cholangiopancreatography (ERCP) for the purpose of relieving patient anxiety. It has been reported that CO_2_ accumulated during sedation causes an arousal reaction, so how to normalize CO_2_ during sedation can be improved by administration of the minimum necessary sedative.

Nasal High Flow oxygen therapy (NHF) uses a mild positive pressure load that improves carbon dioxide washout and reduces rebreathing to improve respiratory function and therefore is widely used to prevent hypoxemia and hypercapnia. In this study, we will investigate whether the upper airway patency would be maintained and the hypercapnia and hypoxemia during sedation would be prevented, by applying NHF as a respiratory management method to patients undergoing ERCP under sedation.

**Methods/design:**

In a randomized comparative study of 2 groups, the NHF device use group and the nasal cannula use group, for adult patients who visited the Nagasaki University Hospital and underwent ERCP examination under sedation. For sedation, Dexmedetomidine will be used in combination with and Midazolam and evaluation by anesthesiologist. In addition, as an analgesic, pethidine hydrochloride was administered intravenously. The total dose of the analgesic pethidine hydrochloride used in combination is used as the primary endpoint. As a secondary evaluation item, the percutaneous CO_2_ concentration is evaluated with a TCO_2_ monitor to examine whether it is effective in preventing hypercapnia. Furthermore, we will evaluate the incidence of hypoxemia with a percutaneous oxygen saturation value of 90% or less, and examine whether the use of equipment is effective in preventing the occurrence of hypercapnia and hypoxemia.

**Discussion:**

The purpose of this study was to obtain evidence for the utility of NHF as a potential therapeutic device for patients undergoing an ERCP under sedation, assessed by determining if the incidence rates of hypercapnia and hypoxemia decreased in the NHF device group, compared to the control group that did not use of this device.

## 1. Introduction

In the case of endoscopic retrograde cholangiopancreatography (ERCP) and other relatively invasive examinations and procedures under upper gastrointestinal endoscopy, sedation is widely applied to relieve patient anxiety. In line with the recommended sedative drug described in Guideline released by Japan Gastroenterological Endoscopy Society,^[1]^ the Nagasaki University Hospital Optical Medical Clinic also uses Dexmedetomidine which have less respiratory depression and analgesic effect, to perform procedural sedation. However, sedation during ERCP is often maintained at moderate to deep sedation to reduce patient stress to invasion, and even with Dexmedetomidine, which has the advantage of less respiratory depression, there is always a risk of upper respiratory tract obstruction due to the relaxing action of the large muscles and narrowing of the airway due to repositioning, and there is also the risk of presenting cardiovascular symptoms such as sedation. Interestingly, it has been reported that respiratory complications that occur during intravenous sedation have a higher risk of hypercapnia than hypoxemia.^[2–6]^

Therefore, how to prevent CO_2_ accumulation and hypoxemia due to transient or persistent upper airway obstruction during procedural sedation is extremely important for safe respiratory management. Furthermore, interestingly, it has been reported that CO_2_ accumulated during sedation causes an arousal response. Hsu YW et al Reported that a 5% CO_2_ load evoked an arousal response during sedation of dexmedetomidine, concluding that this is similar to the physiological changes that occur during natural sleep in the olden days.^[7]^ Therefore, how to normalize CO_2_ during sedation is considered to be an important factor for obtaining more stable sedation with the administration of the minimum necessary sedative.

On the other hand, the applied effect of a device called Nasal High flow (hereinafter abbreviated as NHF) that inhales humidified high flow oxygen and air, which is one of the respiratory control methods, is attracting attention. The NHF load machine (trade name AIRVO3), which flows a high flow of oxygen from the nasal cavity, is used for patients with obstructive sleep aspiration syndrome and respiratory disorders, and has a mild positive pressure load (several cmH_2_O) and exhaled CO_2_. Respiratory function is improved by washing out and reducing rebreathing, and it has begun to be widely applied to prevent hypoxemia.^[8–11]^ In our previous study, we confirmed that the use of NHF under sedation has the effect of stabilizing breathing,^[12]^ and found that the combined use of NHF during ERCP under sedation can prevent the accumulation of CO_2_. Interesting findings have been obtained that the total dose of the concomitant analgesic (pethidine hydrochloride) tend to be reduced to obtain the same sedation.^[13,14]^

In this study, we applied NHF as a respiratory management method to patients undergoing ERCP under sedation to prevent the onset of intraoperative hypercapnia and reduce the respiratory workload during sedation. The purpose is to examine whether sedation can be stabilized by reducing the arousal response.

Although there are some studies on the prevention of hypoxemia by performing respiratory management using NHF during ERCP, there are no clinical trials in Japan or overseas to verify whether the sedative effect is stabilized. Upon completion of this study, patients will be able to receive ERCP treatment under safer, less stressful sedation.

Objective: In this study, the Clinical Question asked, “Can NHF stabilize breathing and increase sedation under the same sedative dose?,” “Use NHF at the recommended doses of Dexmedetomidine and Midazolam?” The purpose of verifying the Research Question: Is there a difference in the total dose of pethidine hydrochloride, a concomitant analgesic, if the same SEDLINE sedation and Ramsay scale indicators (minimum and area under the curve [AUC]) are maintained in the group?

Significance: If NHF can be used in combination during sedation with Dexmedetomidine and Midazolam to suppress the arousal response during sedation and stabilize the degree of sedation, the minimum amount of analgesic used with sedatives is necessary. We believe that limited administration of sedative drugs can provide a high-quality systemic management method with fewer side effects.

## 2. Methods/design

### 2.1. Study design

The present study is designed in accordance with the Standard Protocol Items: Recommendations for Interventional Trials and Consolidated Standard of Reporting Trials 2010 guidelines.^[15,16]^ This clinical trial is an open-label, investigator-initiated, single center study on the efficacy of NHF use in patients undergoing ERCP performed under intravenous anesthesia. The Clinical Research Review Board in Nagasaki University approved the study and study protocol. The study is conducted at Nagasaki University Hospital in Japan. The study is registered on the jRCTs. The study was conducted in accordance with the principles of the Declaration of Helsinki and the established best clinical practices of Japan.

### 2.2. Participant recruitment

Participants are recruited from the Nagasaki University Hospital, where the subsequent study also took place. The treating clinical research coordinator provided an explanation of the study to all participants, and these participants signed an informed consent form. This is a randomized control study, comprised of 2 groups of participants. While both groups received intravenous anesthesia during ERCP, 1 group concomitantly used an NHF device. After giving consent, subjects who meet the registration requirements are randomly assignment to 1 of the 2 groups.

### 2.3. Inclusion criteria

The following were inclusion criteria: Age: Adult patients whose age is between 20 and 85 years old at the time of obtaining consent. Gender: Any question. Hospitalization/outpatient: hospitalization only. Patients who have received sufficient explanation for participation in this study, and who have received sufficient written consent from the patient himself/ herself with sufficient understanding.

### 2.4. Exclusion criteria

The exclusion criteria are as follows: Cases of postoperative reconstructed intestinal tract. Continuous administration of oxygen by nasal cannula (home oxygen therapy). Patients who cannot breathe nose. Patients who cannot reduce or discontinue anti thrombotic drugs on the day before the endoscope. Patients with a history of pneumothorax. Patients who are positive for the new coronavirus (SARS-CoV-2) PCR test. Patients with a history of bronchial asthma attack or severe asthma. Other patients who are determined to be inappropriate as research subjects by the principal investigator.

### 2.5. Study protocol

#### 2.5.1. Overview of treatment (protocol treatment).

In a randomized comparative study of 2 groups, the NHF device use group and the nasal cannula use group, for adult patients who visited the Department of Optical Medicine, Nagasaki University Hospital and underwent ERCP examination and treatment under sedation during hospitalization. Patients who meet the enrollment requirements will be randomly assigned to either the device use group or the nasal cannula use group after obtaining consent (ratio 1:1). Patients are enrolled in patients undergoing ERCP under sedation with Dexmedetomidine. The patient is placed in the examination position on the examination table in the endoscopy room. Patients assigned to the NHF device group inserted a cannula of the NHF device AIRVO3 from both nasal cavities and inhale humidified air and 100% oxygen (% FiO_2_ 0.30–0.40) at a flow rate of 50 to 70 L/min. If the percutaneous oxygen saturation, which is an index of oxygenation, shows 90% or less during sedation, increase the proportion of 100% oxygen to increase the oxygen concentration. Patients assigned to the normal nasal cannula use group inhale 100% oxygen at a flow rate of 3 to 5 L/min (FIO_2_ 0.32–0.40). In the nasal cannula group, if the SpO_2_ value is 90% or less during sedation, increase the inhaled flow rate to 5 L/min to increase the inhaled oxygen concentration.

Sedation is performed using Dexmedetomidine and Midazolam in combination, and the recommended dose in the guideline is administered according to the administration protocol of the usual medical treatment by ERCP at Nagasaki University Optical Clinic. After changing the position, attach various monitors and measure the basic value of each monitor. Dexmedetomidine is administered at 6 μg/kg/hour for 5 minutes as the initial loading administration, and midazolam is administered at 0.05 mg/kg at the same time, and pethidine hydrochloride 1 A (35 mg) is further administered as an analgesic. After completing the administration of Dexmedetomidine at 6 μg/kg/hour for a total of 10 minutes as the initial load administration, change the Dexmedetomidine to 0.7 μg/kg/hour and continue, carefully shifting to sedation maintenance and optimal sedation (30–50 for SEDLINE, 5–6 for RAMSAY SCORE). When the patient falls asleep, insert the scope and start the procedure. At this point, if the patient body movement is observed, add 0.05 mg/kg of midazolam.

When additional intravenous pethidine hydrochloride is administered, the administration unit is 17.5 mg, and when used in combination with Dexmedetomidine, the Ramsay Score is 5 (slow response to light tapping to the drowsiness between the eyebrows or strong auditory stimulus) to 6 (no response to stimulus). In order to maintain a sedation level of about 30 to 50, pethidine hydrochloride is administered 17.5 mg intermittently, and a total of 105 to 140 mg should be administered. As a guide, administer as needed to maintain the same degree of sedation. The additional dose of pethidine hydrochloride should be 17.5 mg per dose, and the respiratory depression caused by the administration of pethidine hydrochloride in a short period of time should be carefully determined.

(Reference: Ramsay Score score 5 (slowly responds to somnolence between the eyebrows or strong auditory stimuli) and 6 (no response to stimulus))

Using SEDLINE as a sedation monitor, continuous measurement of sedation is performed, an electroencephalogram electrode is attached to the forehead, recorded in the device, and externally output to a USB medium after the test is completed.

A normal sphygmomanometer, electrocardiogram, and percutaneous oxygen saturation measuring probe used during examination and treatment are attached to the finger, and a percutaneous CO_2_ concentration sensor is attached to the precordium to continuously measure vital signs. To continuously and non-invasively assess blood CO_2_ levels, we use percutaneous CO_2_ levels that are unaffected by inhalation of high-flow air with nasal cannula on. It was

Percutaneous CO_2_ concentration is measured by continuously measuring percutaneous carbon dioxide concentration by attaching an electrode for Transcutaneous CO2 monitor (manufactured by Radiometer) to the anterior chest, and measuring data together with percutaneous oxygen saturation and respiratory rate. Reads from the anesthesia record on the electronic medical record that recorded the value of the patient monitoring monitor manufactured by Nihon Kohden.

Respiratory rate detection uses the respiratory detection function of RADICAL7 to continuously record the respiratory rate. In addition, the respiratory rate is evaluated from the impedance change between the electrodes of the 3-pole standard lead of the electrocardiogram in case the measured value becomes inaccurate due to noise mixed under the influence of NHF.

### 2.6. Adverse events

In this study, the NHF device was secured to the patient via a nasal cannula, and this might have felt uncomfortable. Moreover, an electrode for transcutaneous CO_2_ (PtcCO2) concentration measurements was attached to the anterior chest for both the NFH group and non-NHF group. For participants with sensitive skin, this might have caused temporary redness. When these adverse events occurred, care was taken to prevent undue harm to the research subjects.

### 2.7. Outcome

The primary outcome is; total dose of pethidine hydrochloride, an analgesic used in combination.

Secondary outcomes are: Occurrence rate of marked hypercapnia showing a maximum percutaneous carbon dioxide concentration of 60 mm Hg or more (corresponding to PaCO_2_ > 55 mm Hg) during intravenous anesthesia, Area under the curve (AUC) of percutaneous carbon dioxide concentration per unit time during intravenous anesthesia, Duration of moderate hypercapnia showing a maximum percutaneous carbon dioxide concentration of 50 mm Hg or more (equivalent to PaCO_2_ > 45 mm Hg) during intravenous anesthesia, Incidence of hypoxemia with percutaneous oxygen saturation of 90% or less during intravenous anesthesia, Respiratory rate using Radical 7, Respiratory rate from changes in impedance of ECG electrodes, Total dose of sedative (Dexmedetomidine and Midazolam), dose per anesthesia time, Sedation level using SEDLINE, Ramsay scale, which is an objective evaluation.

### 2.8. Efficacy

We evaluated the efficacy of the investigational device on a number of parameters, including: changes in CO_2_ concentration during treatment, the rate of occurrence of marked hypercapnia in which the maximum value of PtcCO_2_ concentration is 60 mm Hg or more (equivalent to PaCO_2_ > 55 mm Hg), and the duration of moderate hypercapnia as defined by a PtcCO_2_ concentration of 50 to 60 mm Hg (equivalent to PaCO_2_ > 45 mm Hg). Additionally, the incidence of hypoxemia was evaluated as defined by a transcutaneous oxygen saturation value of 90% or lower.

### 2.9. Safety

The safety evaluation indices of this clinical trial are as follows: adverse events are any undesired or unintended signs (including abnormal laboratory values, abnormal vital signs), symptoms, or illnesses that occur between the start of medical device (NHF) use and the end of the last observational study. This does not matter whether the study has a causal relationship. Symptoms and diseases occurring before the use of medical devices are treated as complications and not adverse events. However, if the complications worsen after the date of starting medical device use, they will be treated as adverse events, and the day on which the deterioration is confirmed will be the date of occurrence of the adverse events.

### 2.10. Data collection and management

The assignment table and input table used in this study were created with Research Electronic Data Capture (REDCap). The study was conducted after allocating the registered patients, and the data of all items in the medical record collected in the study were assigned to the researcher assigned the ID entered by physician, co-doctor and coworker. The Principal Investigator or Co-Researcher approved the input observation/ inspection/ evaluation data of each research subject immediately after confirming the content. For the data entered in the case report, the Principal Investigator and the Clinical Research Center Data Management staff perform a visual check and a logical check. Consequent to each check, if there are any problems or doubts in the data, the principal investigator, or the research coordinator is contacted. The case is fixed by performing data lock on the case when the issue has been resolved, and any modifications have been completed. If there is an error that needs to be corrected after the case is locked, the data management staff is responsible for overseeing this process.

In this study, monitoring will be carried out in accordance with the research plan and monitoring procedures to ensure that the research is being conducted properly.

### 2.11. Sample size

#### 2.11.1. 118 cases (59 cases per group).

The sample size was calculated using the total dose unit (1 unit 17.5 mg) of the concomitant analgesic pethidine hydrochloride, which is the primary endpoint of this study. From an exploratory study conducted in advance, the average total dose of pethidine hydrochloride in the NHF-using group and the low flow oxygen group was 2.774 units and 3.933 units, respectively.^[14]^ From this, it was hypothesized that the total unit of administration of pethidine hydrochloride follows a Poisson distribution with exp (1.02) = 2.774 in the NHF-using group and exp (1.37) = 3.933 in the non-NHF use group. Simulations were performed using a Poisson regression model with the objective variable as the total dose unit of pethidine hydrochloride and the explanatory variables as the group. When the number of repetitions was 1000 and the bilateral significance level was 5%, the minimum sample size with a power of 90% or more was 53 cases per group. Assuming a dropout rate of 10%, the target number of cases in group 1 was 59, and that in group 2 was 118.

### 2.12. Statistical analysis

All research subject groups assigned in this study are referred to as ITT (Intention to treat). Of the ITTs, the research subjects for whom data on the primary endpoints have been obtained are referred to as full analysis set.

The total dose of pethidine hydrochloride, a concomitant analgesic, is tabulated for each group. Using a Poisson regression model with the total dose unit of pethidine hydrochloride as the objective variable and the group as the explanatory variable, the ratio of the mean total dose units for each group, the 95% confidence interval based on the Wald statistic, and the bilateral *P* value are calculated. The significance level is set to 5%, and when the 2-sided *P* value falls below the significance level, it is judged to be statistically significant. In addition, the median difference and 95% confidence interval for each group are calculated using a quantile regression model with the total dose of the combined analgesic pethidine hydrochloride as the objective variable and the group as the explanatory variable.

Summarize each group appropriately according to the scale of each endpoint, and use a Poisson regression model with each secondary endpoint as the objective variable and the group as the explanatory variable, as in the case of the primary endpoint, for each group. Calculate the mean total unit ratio, 95% confidence interval and bilateral *P* values based on the Wald statistic. The significance level is set to 5%, but the multiplicity is not adjusted and the *P* value has no definitive meaning.

## 3. Discussion

The goal of using an NHF device, during an ERCP under intravenous anesthesia, is to prevent not only acute hypercapnia, but also hypoxemia. The clinical conditions resulting from a transient upper airway obstruction that can occur during intravenous anesthesia could be prevented or ameliorated by the use of an NHF device. It has been reported that the frequently used intravenous anesthesia during an ERCP is associated with the higher occurrence rate of respiratory depression.^[17]^ Furthermore, it has been reported that respiratory complications that occur during intravenous anesthesia have a higher risk of hypercapnia than hypoxemia.^[2–6]^ Therefore, the prevention of hypercapnia could be important factor for achieving safe respiratory management during procedural sedation for ERCP.

This is an verifiable study aims to test that there would be a difference in the total dose of pethidine hydrochloride, a concomitant analgesic, if the same SEDLINE^®^ sedation and Ramsay scale indicators (minimum and AUC) are maintained in the same degree in both group. The sample size of 118 cases (59 cases per group) is calculated using the total dose unit (1 unit 17.5 mg) of the concomitant analgesic pethidine hydrochloride, which is the primary endpoint of this study. From an exploratory study conducted in advance, the average total dose of pethidine hydrochloride in the ERCP-using group and the non-ERCP group was 2.774 units and 3.933 units, respectively. From this, it was hypothesized that the total unit of administration of pethidine hydrochloride follows a Poisson distribution with exp (1.02) = 2.774 in the ERCP-using group and exp (1.37) = 3.933 in the nonuse group. Simulations were performed using a Poisson regression model with the objective variable as the total dose unit of pethidine hydrochloride and the explanatory variables as the group. When the number of repetitions was 1000 and the bilateral significance level was 5%, the minimum sample size with a power of 90% or more was 53 cases per group. Assuming a dropout rate of 10%, the target number of cases in group 1 was 59, and that in group 2 was 118.

The primary endpoint of this study is; total dose of pethidine hydrochloride, an analgesic used in combination. In addition, occurrence rate of marked hypercapnia showing a maximum percutaneous carbon dioxide concentration of 60 mm Hg or more (corresponding to PaCO_2_ > 55 mm Hg) during intravenous anesthesia, AUC of percutaneous carbon dioxide concentration per unit time during intravenous anesthesia, duration of moderate hypercapnia showing a maximum percutaneous carbon dioxide concentration of 50 mm Hg or more (equivalent to PaCO_2_ > 45 mm Hg) during intravenous anesthesia, incidence of hypoxemia with percutaneous oxygen saturation of 90% or less during intravenous anesthesia, respiratory rate using monitoring device, respiratory rate from changes in impedance of ECG electrodes, total dose of sedative (Dexmedetomidine and Midazolam), dose per anesthesia time, sedation level using monitoring device and Ramsay scale, which is an objective evaluation will be also be assessed as a secondary endpoint. Recently Hung KC et al suggested the efficacy of high flow nasal oxygenation for reducing the risk of hypoxemia in patients receiving elective gastrointestinal endoscopic procedures under sedation.^[18]^ Zhang YX et al^[19]^ also indicated that efficacy of NHF not only to reduce the incidence of hypoxemia but also reduce the requirements for airway intervention during sedated digestive endoscopy procedures, especially in patients at low risk for hypoxemia. Furthermore, we have recently suggested that the application of NHF did not result in a significant reduction in the occurrence of marked hypercapnia during an ERCP procedure with sedation.^[14]^ Therefore, this randomized clinical trial might obtain new insight to evaluate the efficacy of NHF on respiratory management. Schumann et al reported that the availability to use NHF during sedation in an endoscopy suite reduced the requirement for general anesthesia to perform complex endoscopic procedures.^[20]^ NHF reduces the re-breathing of expired CO_2_ from the anatomical dead space, which allows for maintained gas exchange at a lower minute ventilation.^[21,22]^ Therefore, patients can achieve the same alveolar ventilation with a reduced workload for the respiratory muscles.^[23]^ The most recent our study indicated that during sedation with propofol, NHF without supplemental oxygen attenuates CO_2_ retention and reduces the respiratory rate.^[12]^ Pinkham M^[22]^ also suggested that the experimental data demonstrates the rise in pressure with the increase of the cannula size and flow rate. The findings suggest that NHF can improve ventilation during procedural sedation for relatively invasive upper gastrointestinal endoscopy procedures, such as an ERCP, and also lower gastrointestinal endoscopy procedures, which may reduce the risk of complications related to hypoventilation.

## Acknowledgments

The authors would like to thank our colleagues and staff at the Dental Anesthesiology, Gastroenterology and Hepatology Department of Nagasaki University Hospital for their support.

## Author contributions

**Conceptualization:** Takao Ayuse, Shinji Kurata, Tomotaka Mori, Shohei Kuroda, Taiga Ichinomiya, Rintaro Yano, Gaku Mishima, Eisuke Ozawa, Stanislav Tatkov, Shuntaro Sato, Nakao Kazuhiko, Tetsuya Hara.

**Formal analysis:** Shohei Kuroda, Shuntaro Sato.

**Supervision:** Takao Ayuse, Nakao Kazuhiko, Tetsuya Hara.
